# Draft Genome Sequence of the Human-Pathogenic Fungus *Scedosporium boydii*

**DOI:** 10.1128/genomeA.00871-17

**Published:** 2017-09-14

**Authors:** Ludovic Duvaux, Jason Shiller, Patrick Vandeputte, Thomas Dugé de Bernonville, Christopher Thornton, Nicolas Papon, Bruno Le Cam, Jean-Philippe Bouchara, Amandine Gastebois

**Affiliations:** a Groupe d’Etude des Interactions Hôte-Pathogène (EA 3142), GEIHP, Université d'Angers and Université de Brest, Université Bretagne-Loire, Angers, France; b UMR 1345 IRHS, INRA, AGROCAMPUS-Ouest, Université d’Angers, SFR 4207 QUASAV, Beaucouzé, France; c Laboratoire de Parasitologie-Mycologie, Centre Hospitalier Universitaire, Angers, France; d Université François Rabelais de Tours, EA 2106, Biomolécules et Biotechnologies Végétales (EA2106), Tours, France; e Biosciences, University of Exeter, Exeter, United Kingdom

## Abstract

The opportunistic fungal pathogen *Scedosporium boydii* is the most common *Scedosporium* species in French patients with cystic fibrosis. Here we present the first genome report for *S. boydii*, providing a resource which may enable the elucidation of the pathogenic mechanisms in this species.

## GENOME ANNOUNCEMENT

The filamentous fungus *Scedosporium boydii* (formerly *Pseudallescheria boydii*) is a soil saprotroph. As an opportunistic pathogen, it causes infections in humans ranging from localized infections, such as subcutaneous mycetoma (1), to fatal disseminated infections in immunocompromised patients, particularly after lung or heart-lung transplantation (2). *Scedosporium boydii* has received increasing attention since it was recognized as a significant pathogen for patients with cystic fibrosis (CF) (3). *Scedosporium boydii* was long considered the sexual state of *Scedosporium apiospermum*, but a multifaceted study has demonstrated that *S. boydii* and *S. apiospermum* are distinct species, and a new species, *Scedosporium dehoogii*, was therefore proposed (4). Since then, several new species have been assigned to the *Scedosporium* genus, which now comprises 10 species (A. Ramirez-Garcia, A. Pellon, A. Rementeria, I. Buldain, E. Barreto-Berguer, R. Rollin-Pinheiro, J. V. de Meirelles, S. Ranque, V. Havlicek, P. Vandeputte, Y. Le Govic, J.-P. Bouchara, S. Giraud, S. Chen, J. Rainer, A. Alastruey-Izquierdo, M. T. Martin-Gomez, L. M. López-Soria, J. Peman, C. Schwarz, A. Bernhardt, K. Tintelnot, J. Capilla, A. M. Vicente, J. Cano-Lira, M. Nagl, M. Lackner, L. Irinyi, W. Meyer, S. de Hoog, F. L. Hernando, submitted for publication). The most frequent species associated with CF differ between countries: *Scedosporium apiospermum* is particularly common in CF in Germany (5), and *Scedosporium aurantiacum* in Australia (6), representing 55% and 50% of all *Scedosporium* isolates, respectively, while *S. boydii* is the predominant species in French CF patients (62% of the isolates) (7). Genomes of *S. apiospermum* and *S. aurantiacum* species have been published recently (8, 9), and here we report the genome of *Scedosporium boydii* IHEM 23826, isolated on 26 November 2009 at the University Hospital of Angers from respiratory secretions of a CF patient. This is the first genome report for this species.

Genomic DNA was sequenced on an Illumina MiSeq platform, generating a total of 2.25 million paired-end reads of 300 bp in length at the ANAN technical platform of the SFR Quasav (INRA, Université d’Angers). We used Trimmomatic (version 0.36) (10) to clean the reads before genome assembly with the SPAdes pipeline (version 3.9.0) (11). The genome assembly was 43.3 Mb in length, consisting of 587 contigs ranging from 0.5 to 520 kb in size. Using the BUSCO pipeline (version 1.1b1) (12), we estimated the completeness of our genome to be 98.7% (1,419 ultraconserved genes found out of 1,438), which is very similar to the 98.82% (1,421 genes found) of the genome of *S. apiospermum* strain IHEM 14462 (8). The GC content and *N*_50_ value were 50.69% and 151,073 bp, respectively.

Gene prediction was performed using Augustus (version 3.2.3) (13). Training files used for the gene prediction were produced with the BRAKER1 program (14) for the closely related fungus *S. apiospermum* (strain IHEM 14462), using RNA sequencing data from a number of different growth conditions (our unpublished results). Using this approach, 11,633 genes were predicted in the *S. boydii* genome. This is comparable to *S. apiospermum* strain IHEM 14462 and *S. aurantiacum* strain WM09.24, with 10,919 and 10,525 predicted genes, respectively (8, 9).

These additional genomic resources will allow comparative genomic analysis to be made among pathogenic *Scedosporium* species and will increase the data available to study the molecular basis of pathogenicity and antifungal drug resistance in these organisms.

### Accession number(s).

This whole-genome shotgun project has been deposited at DDBJ/EMBL/GenBank under the accession number NJFT00000000. The version described in this paper is the first version, NJFT01000000.
